# Lamb Waves Propagation along 3C-SiC/AlN Membranes for Application in Temperature-Compensated, High-Sensitivity Gravimetric Sensors

**DOI:** 10.3390/s130100550

**Published:** 2013-01-02

**Authors:** Cinzia Caliendo, Arnaldo D'Amico, Fabio Lo Castro

**Affiliations:** 1 Istituto dei Sistemi Complessi, ISC-CNR, Via Salaria Km. 29.300, Monterotondo Scalo, 00015 Roma, Italy; 2 Dipartimento di Ingegneria Elettronica, Università di Tor Vergata, Via del Politecnico 1, 00133 Roma, Italy; E-Mail: damico@eln.uniroma2.it; 3 Istituto di Acustica e Sensoristica “Orso Mario Corbino”, IDASC-CNR, Via del Fosso del Cavaliere 100, 00133 Roma, Italy; E-Mail: fabio.locastro@idasc.cnr.it

**Keywords:** AlN, SiC, suspended membranes, S_0_ Lamb mode, thermal stability, sensors

## Abstract

The propagation of the fundamental quasi-symmetric Lamb mode S_0_ travelling along 3C-SiC/*c*-AlN composite plates is theoretically studied with respect to the AlN and SiC film thickness, the acoustic wave propagation direction and the electrical boundary conditions. The temperature effects on the phase velocity have been considered for four AlN/SiC-based electroacoustic coupling configurations, specifically addressing the design of temperature-compensated, enhanced-coupling, GHz-range electroacoustic devices. The gravimetric sensitivity and resolution of the four temperature-stable SiC/AlN composite structures are theoretically investigated with respect to both the AlN and SiC sensing surface. The SiC/AlN-based sensor performances are compared to those of surface acoustic waves and Lamb S_0_ mode mass sensors implemented on bulk conventional piezoelectric materials and on thin suspended membranes.

## 1. Introduction

In recent years AlN piezoelectric thin films have been used to fabricate a wide variety of radio frequency resonators and filters for applications in telecommunication and sensing fields, thanks to their high acoustic wave velocity, good electromechanical coupling coefficient (K^2^), and resistance to severe environmental conditions [1]. Cubic polytype SiC (3C-SiC) substrates have been proven to be suitable for the implementation of AlN-based multilayered devices thanks to some interesting properties such as low mechanical loss, a high surface acoustic wave (SAW) velocity, resistance to chemicals, high hardness, the low lattice mismatch (1%) and a thermal expansion coefficient that closely matches that of AlN. Moreover AlN and SiC show opposite temperature coefficients of delay (TCD), thus allowing the fabrication of temperature-compensated electroacoustic devices for the proper AlN and SiC films thickness. Large-area low-cost 3C-SiC(100) and (111) films on Si(001) and (111) substrates are commercially available [2,3] for limited thickness ranges (up to 1 and 20 μm thick). Highly *c*-axis oriented AlN films have been grown on 3C-SiC films by using different techniques, such as metalorganic vapor phase epitaxy (MOVPE) [4], low pressure metalorganic chemical vapor deposition (LP-MOCVD) [5], and reactive magnetron sputtering [6]. The latter technique can also be employed to grow metal films (such as Pt or Mo) to be patterned for the implementation of interdigital transducers (IDTs) on the AlN free surface or at the SiC/AlN interface. The Si/SiC/AlN-based structures can take advantage from the anisotropic bulk Si micromachining technique to etch away the silicon lying under the SiC film and leave the SiC/AlN/IDTs as a freely suspended membrane.

Recently, the fundamental quasi-symmetric Lamb wave mode S_0_ propagating along 3C-SiC/c-AlN thin membranes has been theoretically investigated, confirming the *c*-axis oriented AlN thin films on epitaxial 3C-SiC layers as an enabling technology for high-frequency and high-*Q* Lamb wave devices [7]. In the present study the effect of the temperature on the propagation of the S_0_ Lamb mode along 3C-SiC/c-AlN composite plates is studied. Our goal is to provide the design parameters required for gravimetric sensors able to work in harsh environments and showing remarkable performance features such as high-frequency operation, applicability of temperature-compensation techniques, and enhanced-K^2^.

## 2. Theoretical Investigation of the Acoustic Waves Propagation in 3C-SiC/c-AlN

In 3C-SiC/c-AlN-based electroacoustic devices the anisotropy of the SiC layer strongly affects the characteristics of the acoustic waves' (AWs) propagation rather than the c-AlN that is isotropic in the c plane. Hence the Lamb modes propagation characteristics are influenced by factors other than by the AlN layer thickness such as the SiC thickness, cut and propagation direction. The AWs' propagation along 3C-SiC/c-AlN layered substrates was investigated by exploiting the numerical code developed by Adler and coworkers at McGill University (Montreal, Canada): a detailed description of the propagation of surface and plate acoustic waves in layered structures can be found in Farnell and Adler's work [8]. The velocity calculations were performed under the hypothesis of lossless materials, and assuming the single crystal AlN and 3C-SiC material constants available in the literature [9,10]. The SiC/AlN composite plate was considered to be an infinite plate in the *x* and *z* directions, being *x* the AW propagation direction.

### 2.1. Lamb Modes

Two sets of waves propagate along a 3C-SiC plate with finite thickness: the symmetric Lamb waves, whose particle displacements are symmetric about the neutral plane of the plate, and the antisymmetric Lamb waves, whose displacements have odd symmetry about the neutral plane. For sufficiently thin plate only two waves exist: the fundamental symmetric and antisymmetric modes, S_0_ and A_0_[11]. The A_0_ mode is easily identified by its reducing velocity as the plate thickness approaches zero. The S_0_ mode has a weak dispersion region, for small plate thicknesses, where its velocity approaches ${\text{v}}_{\text{s}}\sqrt{2}$ where v_s_ is the shear bulk wave in an unbounded propagating medium. With increasing the plate thickness, the two modes asymptotically reach the SAW velocity in the substrate. Higher order modes exist only starting from a minimum plate thickness value (the cut off) and their velocity asymptotically reach the slow bulk shear velocity in the substrate with increasing plate thickness. A *composite plate* consisting of two different materials, *i.e.*, 3C-SiC/c-AlN plate, has no symmetry about the central plane and the modes are no longer rigorously separated into symmetric and antisymmetric sets. It is intended that the two fundamental modes, arbitrary cited S_0_ and A_0_ modes in the present paper, have to be understood as *quasi-symmetric* and *quasi-antysymmetric* modes [12].

**Figure 1.**
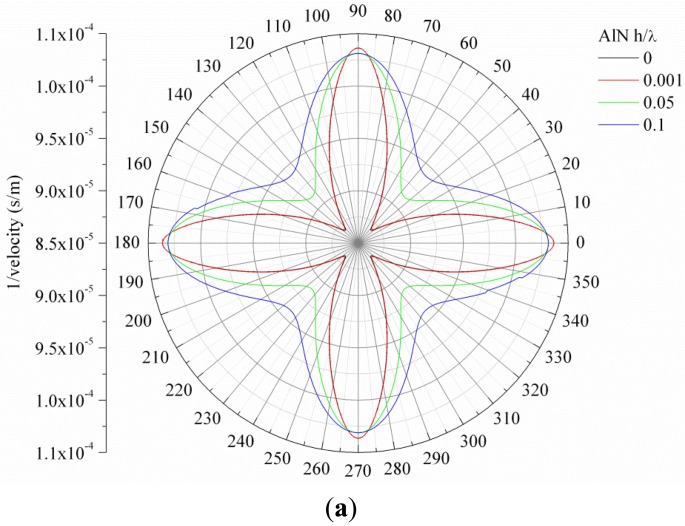

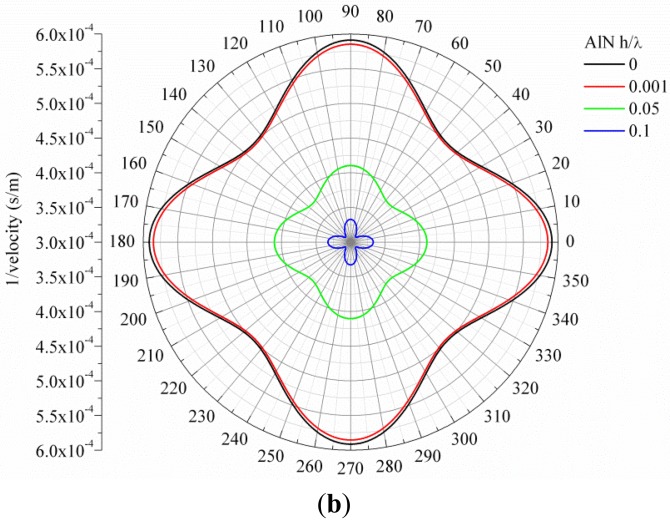
Slowness curves. (**a**) Slowness curves of the S_0_ mode propagating along 3C-SiC plate (0.1 h/λ thick) covered by an AlN layer; (**b**) slowness curves of the A_0_ mode propagating along 3C-SiC plate (0.1 h/λ thick) covered by an AlN layer. In both cases the AlN h/λ (0, 0.001, 0.05 and 0.1) is the running parameter.

Figure 1(a,b) show, as an example, the slowness curve of the S_0_ and A_0_ modes propagating along 3C-SiC/c-AlN composite plate for SiC thickness-to-wavelength-ratio h/λ_SiC_ = 0.1 and AlN normalized thicknesses h/λ_AlN_ = 0, 0.001, 0.05 and 0.1.

For both the <100> and <110> directions of the (001) plane of 3C-SiC, the velocity of the two modes asymptotically reaches the velocity of the SAW in the piezoelectric layer with increasing AlN normalized thickness, h/λ_AlN_. With increasing the SiC layer thickness, the velocities of the two fundamental modes asymptotically converge to the velocity of the first Rayleigh-like mode propagating along these two structures. For propagation directions different from 0° and 45°off x axis in the (001) plane, the phase and group velocity of the propagating modes are not collinear and the direction of energy transport deviates from the phase velocity vector by an angle, the power flow angle (PFA), that depends on the substrate anisotropy and on the deviation angle ϑ with respect to the selected wave vector direction ϑ_0_. Referring to Figure 1(a,b), the phase velocity vector is the radius-vector of the curve drawn from the origin: it determines the wave propagation direction. The direction of the group velocity vector is normal to the curve at the point of intersection of the curve with the wave propagation vector. As it can be seen in Figure 1(a,b), the phase and group velocities are thus collinear for ϑ_0_ = 0° and 45°: for deviation from these directions, the PFA is not null and the resulting beam steering vanishes the practical interest of the device [13,14].

**Figure 2.**
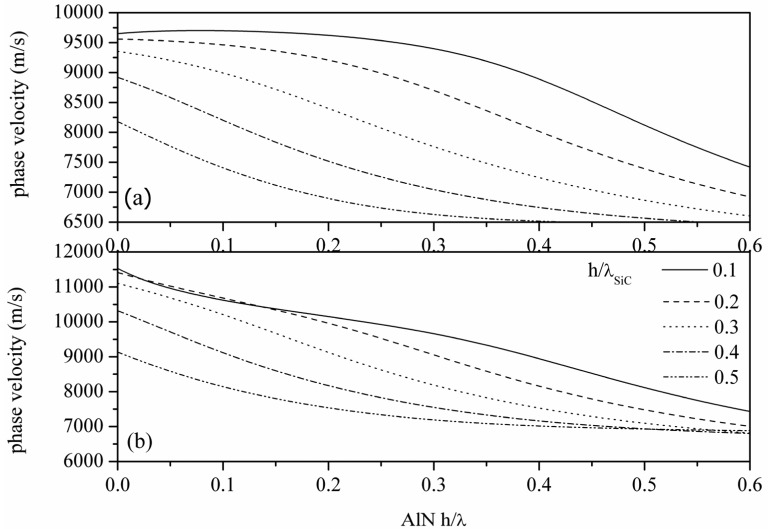
The dispersion curves for the S_0_ Lamb mode propagating (**a**) along c-AlN/SiC(001)<100> and (**b**) along c-AlN/3C-SiC(001)<110>.

Figure 2(a,b) show the calculated phase velocity dispersion curves of the S_0_ mode propagating along the c-AlN/SiC(001)<100> and <110>, for different h/λ_SiC_ values. As it can be seen from Figure 2, higher phase velocities can be reached for AWs propagating along the <110> direction with respect to the waves propagating along <100>. For h/λ_AlN_ ≪ 1, the velocity decreases as the SiC thickness increases; for the same h/λ_SiC_ value, the velocity decreases with increasing the h/λ_AlN_ value. For large AlN and SiC thicknesses, the top and bottom surfaces of the plate become essentially decoupled and the velocity in the plate decreases, matching that of the *Rayleigh-like* mode in AlN/SiC.

The weak dispersion and the high velocity of the S_0_ mode (for h/λ_AlN_ ≪ 1) reduces the AlN thickness uncertainty and enables GHz range (from 6 to 10 GHz) resonators with IDTs' line-width resolution from 0.4 to 0.25 μm.

### 2.2. The Electroacoustic Coupling Coefficient, K^2^

The performances of an electroacoustic device depend on the materials chosen and on the transduction configuration adopted. To find the optimum device configuration, the characteristics of the Lamb mode propagation and transduction are studied as a function of the SiC and AlN layer thickness, the ground electrode and IDTs location with respect to the AlN film surface.

An ideal electric conductor, placed at one or both the surfaces of the piezoelectric layer, shorts the tangential component of the electric field, thus affecting its distribution into the layer, and resulting in a decrease in the acoustic phase velocity. The electroacoustic coupling coefficient, K^2^, is based on the computation of the difference between the phase velocity of the AW propagating along a free piezoelectric surface and that along a metalized piezoelectric surface. Only the electric loading from the IDTs and ground electrode is taken into account, omitting the mass loading effect due to their finite thickness: the IDT metal pattern, as well as the ground electrode, is considered ideal, *i.e.*, perfectly conducting and infinitely thin.

The electroacoustic coupling coefficient, K^2^, is a parameter of primary importance for the design of AW devices as only K^2^ of the total input of the electrical energy is converted to the elastic energy of the AW. For AW propagating along layered structures, the K^2^ is frequency dispersive and depends on the type and orientation of the piezoelectric material, on the propagation direction and on the electrical boundary conditions. K^2^ can be approximated as:

(1) $$K^{2}=2\cdot \frac{v_{ph}^{f}-v_{ph}^{m}}{v_{ph}^{f}}$$

where $v_{ph}^{f}$ and $v_{ph}^{m}$ are the phase velocities along the electrically open and shorted surfaces of the AlN film. The $v_{ph}^{m}$ is obtained by the insertion of a perfectly conductive and infinitesimally thin metal film at the interfaces where the IDTs and the ground plane are located.

**Figure 3.**
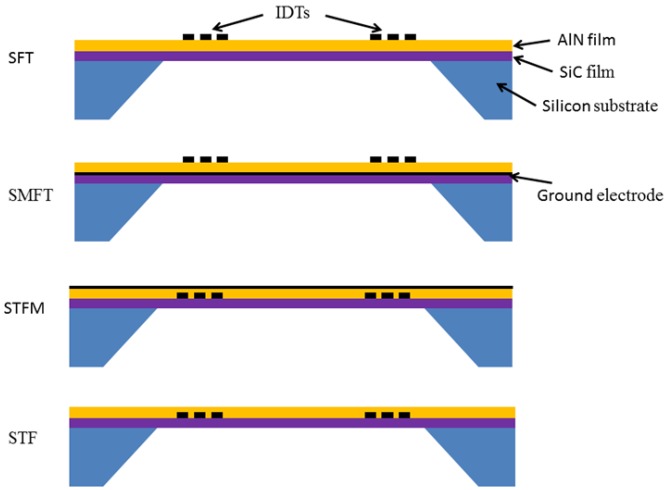
The four coupling configurations.

In the AlN/SiC composite plate, four piezoelectric coupling configurations can be obtained by placing the IDT's at the substrate/film interface (Substrate/Transducer/Film, STF), at the film surface (Substrate/Film/Transducer, SFT), farther including a ground electrode opposite the IDTs (Substrate/Transducer/Film/Metal and Substrate/Metal/Film/Transducer, STFM and SMFT). The four coupling configurations are shown in Figure 3.

**Figure 4.**
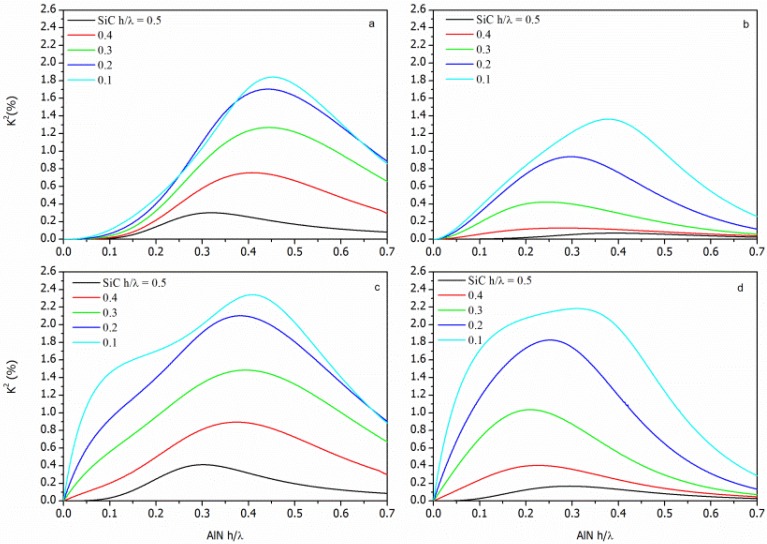
The K^2^ dispersion curves of the (**a**) STF, (**b**) SFT, (**c**) STFM, and (**d**) SMFT coupling configurations on c-AlN/SiC(001)<100> substrates; the h/λ_SiC_ (from 0.1 to 0.5) is the running parameter.

**Figure 5.**
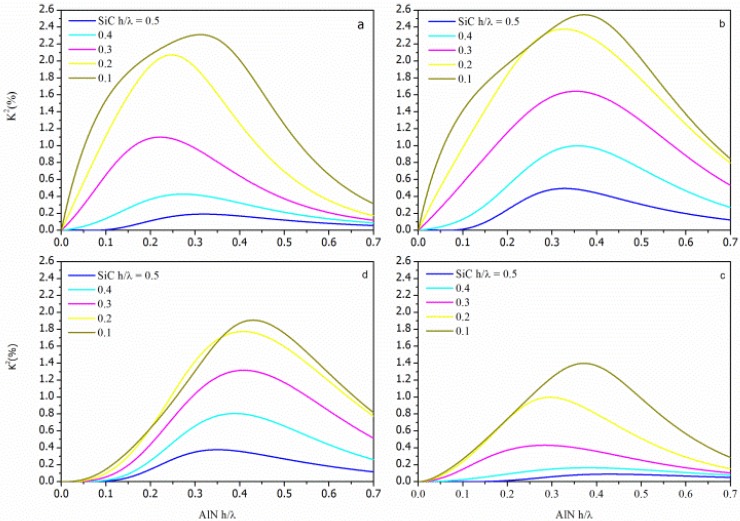
The K^2^ dispersion curves of the (**a**) SMFT, (**b**) STFM, (**c**) SFT, and (**d**) STF coupling configurations on c-AlN/SiC(001)<110> substrates; the h/λ_SiC_ (from 0.1 to 0.5) is the running parameter.

The *K^2^* dispersion curves of the four configurations were evaluated by the relations $K_{\text{SFT}}^{2}=2\cdot \frac{v_{ph}^{SF}-v_{ph}^{\text{SFM}}}{v_{ph}^{SF}}$, $K_{\text{SMFT}}^{2}=2\cdot \frac{v_{ph}^{\text{SMF}}-v_{ph}^{\text{SMFM}}}{v_{ph}^{\text{SMF}}}$, $K_{\text{STF}}^{2}2=2\cdot \frac{v_{ph}^{SF}-v_{ph}^{\text{SMF}}}{v_{ph}^{SF}}$ and $K_{\text{STFM}}^{2}=2\cdot \frac{v_{ph}^{\text{SFM}}-v_{ph}^{\text{SMFM}}}{v_{ph}^{\text{SFM}}}$ for the SFT, SMFT, STF and STFM configurations, respectively, being the SiC and the AlN layers represented as the Substrate and the Film, respectively. The K^2^ dispersion curves (K^2^ vs h/λ_AlN_) of each AlN/SiC-based configuration were calculated for different h/λ_SiC_ values and for the <100> and <110> propagation directions. The calculated data addressing the AlN/SiC(001)<100> and AlN/SiC(001)<110> are shown in Figures 4 and 5, being the h/λ_SiC_ the running parameter.

For acoustic waves propagating along layered structures the achievable K^2^ can be larger than that of the individual piezoelectric material (K^2^ = 0.28% for AlN). For surface acoustic waves (SAWs) propagating along the free surface of the piezoelectric semi-infinite substrate the energy localization occurs within a thickness of a wavelength, while the Lamb wave's energy is predominantly confined in the piezoelectric layer whose thickness is less than a wavelength. Moreover, the K^2^ dispersion curve is strongly affected by the electrical boundary conditions that influence the potential depth profile: K^2^ reaches high values when a strong electric field is induced in the piezoelectric layer, and it decreases as the thickness of the SiC layer increases. The STFM and SMFT structures exhibit the highest K^2^ value due to the strong electric field that can be induced between the IDT and the electrode.

### 2.3. Temperature Coefficient of Delay

The velocity of the AWs is a function of the material constants (mass density, elastic, piezoelectric and dielectric constants) of the propagating medium, and these constants are affected by the temperature changes: as a result the AW velocity is sensitive to the temperature variations of the surroundings. The S_0_ mode phase velocity at different temperatures was theoretically estimated by modifying the mass density and the elastic constants of AlN and SiC according to their temperature coefficients available from the literature. Table 1 lists the SiC and AlN coefficients of thermal expansion (CTE) and the temperature coefficients of elastic constants used in our calculations.

As it can be seen AlN and SiC have opposite sign temperature coefficients of the elastic constants [9,15–21]. The AlN and SiC mass density and elastic constants were evaluated at different temperatures T by modifying their values according to their temperature coefficients in the following manner: ρ(T) = ρ(20 °C)[1 − (α_11_ + α_22_ + α_33_)ΔT] where ρ(T) is the mass density at a certain temperature T, ΔT = T−20 °C, ρ(20 °C) is the mass density at 20 °C and α_ii_ is the linear temperature expansion coefficient (available in the literature). The elastic constants c_ij_ were calculated at different temperatures as follows: c_ij_(T) = c_ij_(20 °C)(1 + Tcc_ij_ΔT), where c_ij_(20 °C) and c_ij_(T) represent the elastic constants at 20 °C and at a certain T, Tcc_ij_ is the temperature coefficient of the elastic constant c_ij_[22]. The temperature coefficient of velocity (TCV) of the four coupling configurations was assessed through the fractional change of the acoustic velocity, [(*v_T_*_°_*_C_* − *v*_20°_*_C_*)/*v*_20°_*_C_*], as a function of T, being *v_20°C_* and *v_T°C_* the S_0_ mode phase velocity at ambient temperature and at a certain temperature T, for T ranging from −20 to 200 °C. The theoretical TCD = α_eff_ − TCV of the four configurations was calculated for different h/λ_AlN_ and h/λ_SiC_ values, being α_eff_ the effective thermal expansion coefficient of the composite plate along the propagation direction, computed applying the model for thermal bimorph actuators [23]. A temperature compensated structure is obtained when the temperature coefficient of frequency TCf = (1/f)(∂)f/(∂)T) = −TCD is zero. The temperature compensated points (TCPs; are the AlN normalized thicknesses, h/λ_AlN_, at which α_eff_ equilibrates the TCV to form a thermally compensated structure with the first order TCD equal to 0 ppm/°C. Table 2 lists the following parameters: the TCP of each configuration, the K^2^ and the phase velocity of the thermally compensated configurations on c-AlN/3C-SiC(001)<100> and <110> structures.

The listed data are relative to h/λ_SiC_ = 0.1 and 0.2 only, since at higher SiC normalized thicknesses the K^2^ of the corresponding TCPs rapidly decreases.

### 2.4. Gravimetric Sensitivity

The most common sensing application of the electroacoustic devices is based on the gravimetric principle for mass detection. A mass accumulation on the device surface changes the surface density of the propagating medium, hence resulting in an AW velocity shift. If the added mass consists of an ideal thin elastic film that moves synchronously with the oscillating surface, the fractional-velocity-change to added-mass ratio defines the sensor's gravimetric sensitivity S_m_ = [(*v_l_* − *v*)/*v_l_*]/m, being m = ρh, ρ and h the added layer's mass density and thickness, *v* and *v_l_* the unloaded and mass-loaded plate's phase velocity. For a resonator or a delay-line oscillator sensor, the sensitivity can be expressed as S_m_ = [(f − f_0_)/f]/m, being f and f_0_ the perturbed and unperturbed resonant frequency. The theoretical gravimetric sensitivity S_m_ of the thermally compensated AlN/SiC structures was deduced by numerical computer calculation [8] of the S_0_ mode velocity along the plate mass-loaded by a thin lossless SiO_2_ overlay, for different SiO_2_ surface mass density values ρ_SiO2·_h/λ_SiO2_ (ρ_SiO2_ = 2,200 Kg/m^3^). Since the gravimetric detection can occur either on the AlN or SiC plate surface, two normalized gravimetric sensitivity values S_m_/λ were calculated for each configuration. Table 3 lists the sensitivity of the AlN and SiC sensing surfaces of the SiC/AlN composite plate.

These sensitivity values were deduced by the calculation of the relative velocity shift per unit mass loaded for each configuration: the unloaded and mass loaded velocity values, *v* and *v_l_*, were calculated taking into account the electric loading from the IDTs and the ground electrode. The mass loading effect from the IDTs and the ground electrode was not taken into account.

**Figure 6.**
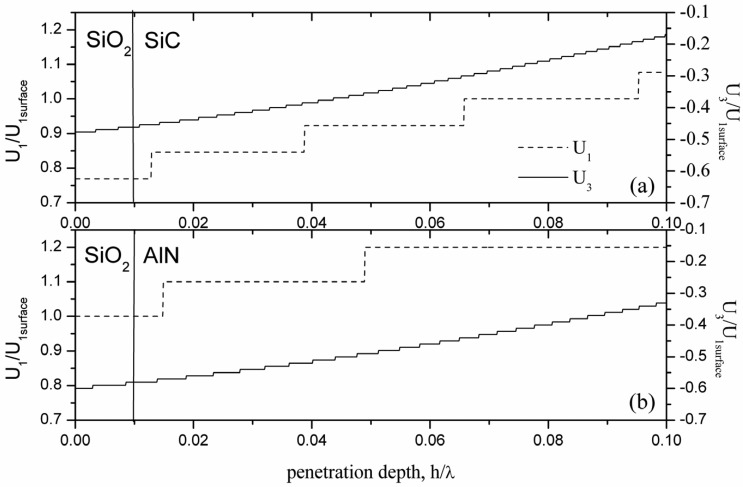
The normalized particle displacement components *vs.* the penetration depth (calculated by 0.0001 h/λ steps) along the (**a**) SiO_2_/SiC and (**b**) SiO_2_/AlN interfaces of the AlN/SiC(001)<110>-SFT plate.

From Table 3 it appears that the gravimetric detection occurring at the AlN or SiC sensing surface of the same plate shows different efficiency. In a composite plate consisting of two different materials, such as AlN/SiC, the AW energy confinement is different close to one or the other plate's surface: the highest gravimetric sensitivity is achieved for the plate sensing surface that transmits an higher amount of acoustic energy to the mass loading layer [24–26]. Figure 6 shows, as an example, the normalized longitudinal and shear vertical particle displacement components $U_{1}\;/\;U_{1}^{\text{AIN surface}}$and $U_{3}\;/\;U_{1}^{\text{AIN surface}}$ *vs* the penetration depth across the SiO_2_/AlN- and SiO_2_/SiC- interfaces of the c-AlN/ SiC(001)<110> plate, being zero the shear horizontal displacement component U_2_. Figure 6 refers to the SFT configuration loaded by a lossless SiO_2_ layer 0.01 h/λ thick, for h/λ_SiC_ = 0.1. From Figure 6 it results that the magnitude of the displacement components at the AlN surface is larger than that at the SiC surface; consequently the relative fraction of the energy density stored in the layer added at the AlN surface is higher than that at the SiC surface, in accordance with the theoretically predicted sensitivity.

Both sides of the four configurations sense the mass and the acoustoelectric loadings from the added layer, except the metalized AlN top surface of the STFM configuration and the SiC bottom surface of the SMFT configuration which can sense only the gravimetric perturbation, as a consequence of the presence of the ground electrode that shorts the electric field associated to the acoustic wave. The acoustoelectric loading produces a decrease in phase velocity and also an increased attenuation for a specific sheet resistance range of the added layer [27]. The fractional phase velocity change induced by the acoustoelectric effect can be isolated from the mass sensor response by subtracting the mass accumulation effect measured by a reference mass sensor [27], such as the SiC-surface of the SMFT or the metalized AlN surface of the STFM.

The mass resolution *R_m_* of a gravimetric sensor is defined as the smallest detectable surface mass per unit area. For a resonator- or for a delay-line oscillator-based sensor, *R_m_* is set by the resonator stability-to-sensitivity ratio:

(2) $$R_{m}=\underset{\Delta f\to \Delta f_{\text{noise}}}{\lim}\overset{\left(\Delta f\;/\;f_{0}\right)}{\;}/\underset{S_{m}}{\;}$$

where *Δf* = *f* – *f*_0_ is the output response, and *Δf_noise_* is defined as three times the frequency random fluctuation, *Δf* = 3Δf_noise_[27]. Temperature-compensated sensors are useful in minimizing the effects of the temperature on the oscillation frequency of the device. *R_m_* has been calculated under the hypothesis of *Δf_noise_* = 1 Hz, at *f*_0_ = *v_TCP_/λ*, being v_TCP_ the phase velocity of the S_0_ mode evaluated at the TCPs; the *R_m_* data are listed in Table 3. As a rule, *S_m_* can be increased by reducing the suspended membrane thickness, but in practical cases the lower limit of the c-AlN and 3C-SiC films thickness is set by the structural properties of the layers. Extremely thin AlN films can show very low K^2^ as a consequence of the poor c-axis orientation of the grains, and extremely thin SiC films on Si substrates could be amorphous and lead to degraded device performances. As an example, for *λ* = 10 μm, the zero-TCD configurations will show *h/λ_SiC_* from 1 to 2 μm, *h/λ_AlN_* from ∼2 to ∼5 μm, S_m_ from ∼230 to ∼385 g/cm^2^, *R_m_* from 8 to 14 pg/cm^2^, f_0_ from ∼750 to ∼900 MHz, and K^2^ from 0.55 to 2.22%.

The gravimetric sensor can also be used for the characterization of film properties such as film thickness and surface area; it is also useful to monitor processes such as thin film deposition or removal, and materials modifications [27]. The mass resolution values *R_m_* listed in Table 3 are referred to 1 Hz of frequency fluctuation, as it is reasonable in ST quartz resonators, just to cite an example. For a thin film device, the frequency fluctuation is higher as a consequence of the polycrystalline nature of the films although highly oriented. Supposing to apply the SiC/AlN-based sensor to monitor the deposition of a thin SiO_2_ layer, a film thickness resolution in the range from 1 to 2 × 10^−12^ m can be obtained under the following hypothesis: *λ* = 2 μm and TCD = 0.5 ppm/°C; in this case the frequency fluctuation is few hundreds of Hz. A lower thickness resolution can be obtained by further reducing the acoustic wavelength.

As shown in Table 3, the AlN sensing side of the structures implemented on SiC(001)<110>/AlN (for *h/λ*_SiC_ = 0.1) seem to be the most promising as they correspond to the highest sensitivity and lowest resolution values. The SiC sensing side is equally highly sensitive for both the two substrates with *h/λ*_SiC_ = 0.2. Among the four configurations, the SFT and SMFT are the most promising as, although they show moderate K^2^, they offer the advantage of a robust technology over the STF and STFM structures for which the IDT strips under the AlN layer can result in thin films cracks. The AlN crystalline quality affects the piezoelectric characteristics of the film and thus of the device performances; thus high-quality AlN film is crucial to achieve temperature compensated structures.

In reference [28] the S_m_ and R_m_ of *SAW* sensors based on both conventional piezoelectric substrates (such as ST-x quartz, yx-, yz-, and yz- 128.086° rot. x-LiNbO_3_) and AlN film on a semiinfinite 3C-SiC substrates were assessed through the fractional change of the acoustic velocity as a function of the mass loading layer thickness. At f_0_ = 100 MHz, the former substrates exhibit good S_m_ (from 62 to 113 cm^2^/g) and remarkable R_m_ (from 0.27 to 0.45 ng/cm^2^), but unfortunately LiNbO_3_ suffers from a very large TCD while the ST*-*quartz shows a very small K^2^. Moreover the quartz and LiNbO_3_ substrates are incompatible with monolithic integration technology, and suffer limited *SAW* velocity (from ∼3,000 to ∼4,000 m/s). The AlN/SiC-based *SAW* configurations, for f_0_ = 100 MHz, show the following properties: S_m_ ∼70 cm^2^/g, R_m_ ∼0.4 ng/cm^2^, K^2^ from 0.71 to 1.18%, TCD from ∼−10 to 5 ppm/°, and phase velocity from ∼5,700 to ∼6,800 m/s. The AlN/SiC-based S_0_ mode configurations show the following properties for *f*_0_ = 100 MHz: *S_m_* from 25 to 52 cm^2^/g, *R_m_* from 0.58 to 1.2 ng/cm^2^, K^2^ from 0.55 to 2.22%, TCD = 0 ppm/°C, and the phase velocity from 7,500 to 10,000 m/s. For the same operation frequency of 100 MHz, the *SAW* sensors are more sensitive than the temperature-compensated S_0_ mode sensors, but their K^2^, phase velocity and TCD are less attractive.

In reference [29] the *S*_0_ mode mass sensor implemented on a GaN suspended membrane is theoretically and experimentally investigated: for *λ* = 16 μm, the sensor operating frequency is 471.3 MHz and its sensitivity is 174 cm^2^/g. For the same wavelength, the AlN/SiC(000) and (0045) sensitivity ranges from 143 to 244 cm^2^/g (for h/λ_SiC_ = 0.1) and from 181 to 256 cm^2^/g (for *h/λ*_SiC_ = 0.2), while the operating frequency ranges from 577 to 621 MHz, and from 471 to 506 MHz, respectively.

In reference [30] the S_0_ mode mass sensitivity (equal to 557 cm^2^/g) of a suspended AlN membrane, 2 μm thick, coated with a hexamethyldisiloxane (HMDSO)-plasma-polymerized thin film (75 nm thick, and showing a mass density of 1,100 Kg/m^3^), was estimated for λ = 12 μm and 888 MHz operating frequency. For the same wavelength, the SiC/AlN-sensor sensitivity ranges from 190 to 325 cm^2^/g (for h/λ_SiC_ = 0.1) and from 242 to 341 cm^2^/g (for *h/λ*_SiC_ = 0.2), while the operating frequency ranges from 770 to 829 MHz, and from 628 to 675 MHz, respectively. The sensitivities and the operating frequency of the AlN/SiC-based sensors are lower than those shown in [30], but they are referred to a temperature compensated device, whose sensitivity and operating frequency can be increased by lowering the acoustic wavelength.

In reference [31] Wingqvist *et al.* demonstrate the remarkable gravimetric sensitivity (1,410 and 500 cm^2^/g) of the SiO_2_ and AlN sides of the temperature compensated S_0_ mode sensor based on thin AlN(2 μm)/SiO_2_(0.79 μm) membranes. The AlN/SiO_2_-based sensor shows an operation frequency f_0_ = 850 MHz and a K^2^ as low as 0.8% for λ = 10.5 μm. These sensitivities are much higher than the highest value obtainable with the AlN/SiC-based sensors for the same wavelength (λ = 10.5 μm). It is important to note that, for equal λ, the thickness of the AlN/SiC plate configurations (from 3.5 to 7.1 μm) is much larger than that of the AlN/SiO_2_ plate (2.79 μm) described in reference [31]. For AlN/SiC plates showing thicknesses comparable with that of the AlN/SiO_2_ plate, (obtainable, for example, by using *λ* = 4 μm), a sensitivity as high as 910 cm^2^/g is achievable on the SiC surface of the AlN(1.7 μm)/SiC(0.8 μm) sensor, for f_0_ ∼2 GHz and K^2^ = 1.77%; a sensitivity as high as 961 cm^2^/g is obtainable on the AlN surface of the AlN(1 μm)/SiC(0.4 μm) plate, for *f*_0_ ∼2.5 GHz and K^2^ =2.17%. Hence, the temperature compensated AlN/SiC plates allow, for comparable layers thicknesses, lower sensitivity but higher operating frequency and K^2^ than those obtainable with the temperature compensated AlN/SiO_2_ plates. Thus, gravimetric sensors showing sensitivity higher than that obtainable with the SiC/AlN-based mass sensors do exist, but at the price of losing the temperature compensation and resistance to harsh environments. Hence the best choice for a practical system requires a compromise between some design parameters (such as the phase velocity, the thermal stability, the K^2^, the *S_m_* and *R_m_*) that concur in the overall device performance evaluation.

The AlN/SiC-based devices can find other possible applications, such as energy harvesting, pressure and temperature sensing, especially at high temperatures and harsh environment, thanks to the AlN and SiC resistance to chemical attacks at elevated temperatures compared to their counterparts such as silicon and ZnO.

## 3. Conclusions

The propagation of the S_0_ Lamb mode along 3C-SiC/c-AlN composite plates have been investigated by theoretical calculation with respect to the AlN and SiC films thickness, acoustic wave propagation direction, electrical boundary conditions and temperature. The phase velocity, the TCD and the K^2^ dispersion curves of four AlN/SiC-based coupling configurations have been theoretically studied specifically addressing the design of temperature-compensated, enhanced-coupling, GHz-range electroacoustic devices. The sensitivity and limit of resolution of gravimetric sensors based on SiC/AlN plates have been theoretically studied with respect to the AlN and SiC sensing surface. The AlN/SiC-based mass sensors are proven to achieve remarkable performances (high sensitivity, low resolution values, thermostability and enhanced coupling efficiency) that are important prerequisite for the design of future devices, based on resonator principles, to be used in the context of chemical, biological and physical quantities detection.

## Figures and Tables

**Table 1.** The SiC and AlN CTE and the temperature coefficients of the elastic constants used in the numerical calculations.

**Material**	$Tcc_{11}^{E}$ **(ppm/°C)**	${\text{Tcc}}_{33}^{\text{E}}$ **(ppm/°C)**	${\text{Tcc}}_{12}^{\text{E}}$ **(ppm/°C)**	$Tcc_{13}^{E}$ **(ppm/°C)**	${\text{Tcc}}_{44}^{\text{E}}$ **(ppm/°C)**	**Ref.**		**CTE (ppm/°C)**	**Ref.**
AlN	−80	−100	−180	−160	−50		9	//c 5.27 ⊥c 4.15	[9]
3C-SiC	71	-	78	-	30		15, 16	3.8	[15]

**Table 2.** The TCP of each configuration, the K^2^ and phase velocity of the thermally compensated configurations.

**Structure**	**h/λ_SiC_**	**Config.**	**TCP**	**K^2^_TCP_ (%)**	**Velocity (m/s)**
c-AlN/SiC(001)<100>	0.1	SFT	0.334	1.31	9,235
		SMFT	0.324	2.16	9,200
		STF	0.331	1.26	9,250
		STFM	0.327	2.11	9,187
	0.2	SFT	0.464	0.55	7,536
		SMFT	0.463	0.83	7,527
		STF	0.473	1.68	7,514
		STFM	0.467	1.93	7,524
c-AlN/SiC(001)<110>	0.1	SFT	0.236	0.94	9,980
		SMFT	0.229	2.17	9,943
		STF	0.233	0.85	9,995
		STFM	0.233	2.11	9,923
	0.2	SFT	0.413	0.76	8,058
		SMFT	0.401	1.25	8,070
		STF	0.413	1.77	8,100
		STFM	0.403	2.22	8,075

**Table 3.** The gravimetric sensitivity S_m_ and the limit of mass resolution R_m_ of the temperature compensated coupling configurations loaded on the AlN or SiC surface with a SiO_2_ film; λ(μm) is the numerical value of the acoustic wavelength expressed in μm.

**Structure**	**SiC (h/λ)**			
	**0.1**		**0.2**	

	a −S_m_/λ(μm) (−cm^2^·g^−1^)	b R_m_ ·λ^2^ (μm) (10^−13^ g·cm^−2^)	c −S_m_/λ(μm) (−cm^2^·g^−1^)	b R_m_ ·λ^2^ (μm) (10^−13^ g·cm^−2^)
c-AlN/SiC(001)<100>				
SFT	2,306; 2,770	1.41; 1.17	2,859; 3,609	1.39; 1.10
SMFT	2,384; 2,714	1.37; 1.20	2,804; 3,598	1.42; 1.11
STF	2,327; 2,775	1.39; 1.17	2,764; 3,624	1.44; 1.10
STFM	2,329; 2,979	1.40; 1.10	2,874; 3,395	1.39; 1.17
c-AlN/SiC(001)<110>				
SFT	3,693; 2,385	0.81; 1.26	3,160; 3,588	1.18; 1.04
SMFT	3,847; 2,351	0.78; 1.28	3,166; 3,567	1.17; 1.04
STF	3,808; 2,380	0.79; 1.27	3,084; 3,640	1.20; 1.02
STFM	3,718; 2,347	0.82; 1.29	3,234; 3,520	1.15; 1.06

a for SiO_2_ h/λ = 0 ÷ 0.04.

b f_0_ = v/λ, being v the velocity of the temperature compensated configuration.

c for SiO_2_ h/λ = 0 ÷ 0.1.
